# Supercritical Fluid Extraction of Lipids from Rowanberry Pomace with Pure CO_2_ and Its Mixtures with Ethanol Followed by the On-Line Separation of Fractions

**DOI:** 10.3390/molecules30040964

**Published:** 2025-02-19

**Authors:** Viive Sarv, Rajeev Bhat, Laura Jūrienė, Renata Baranauskienė, Dalia Urbonavičienė, Pranas Viškelis, Petras Rimantas Venskutonis

**Affiliations:** 1 Polli Horticultural Research Centre, Institute of Agricultural and Environmental Sciences, Estonian University of Life Sciences, 69108 Viljandi, Estonia; viive.sarv@emu.ee; 2 ERA-Chair for Food (By-) Products Valorisation Technologies (VALORTECH), Estonian University of Life Sciences, 51006 Tartu, Estonia; rajeev.bhat@emu.ee; 3 Department of Food Science and Technology, Kaunas University of Technology, LT-50254 Kaunas, Lithuania; laura.juriene@ktu.lt (L.J.); renata.baranauskiene@ktu.lt (R.B.); 4 Biochemistry and Technology Laboratory, Institute of Horticulture LAMMC, LT-54333 Kaunas, Lithuania; dalia.urbonaviciene@lammc.lt (D.U.); pranas.viskelis@lammc.lt (P.V.)

**Keywords:** *Sorbus aucuparia*, berry pomace, supercritical CO_2_ extraction, fractionation, bioactives, volatile compounds

## Abstract

Fruit processing by-products contain various classes of bioactive constituents, which may find applications as ingredients for foods, nutraceuticals or cosmeceuticals. This study explored the fractionation of lipophilic rowanberry pomace extracts isolated with pure supercritical CO_2_ and its mixtures with a co-solvent ethanol by their on-line separation at subcritical conditions. Rowanberry pomace lipids were extracted with supercritical CO_2_ (42.4 MPa, 53 °C) using 0–7% of ethanol, and then fractionated by reducing the first separator’s (S1) pressure to 7 MPa and cooling it to 0, −10 and −20 °C to precipitate the ‘heavier’ fraction (HF). The second separator (S2) was depressurized at ambient temperatures to collect the ‘lighter’ fraction (LF). The yield of the LF increased by decreasing the S1 temperature and increasing the amount of the co-solvent. The concentration of β-carotene was increased in the LF by decreasing the S1 temperature and increasing the co-solvent concentration; at −20 °C it was 66.7% higher than in the non-fractionated extract. The concentrations of tocopherols and phytosterols were also remarkably higher in the LF. In total, 62 compounds were identified in the headspace volatile fraction of the LF, benzaldehyde and benzyl alcohol being the most abundant constituents. In conclusion, fractionation enabled us to obtain fractions with higher concentrations of the selected classes of lipophilic rowanberry constituents.

## 1. Introduction

In the manufacturing of fruit juice, the pressing of fruit generates side-stream residues (called press-cakes or pomaces), which constitute approximately 30% of fruit solids and are regarded as a less valued by-product. Therefore, large amounts of pomace are used rather inefficiently, e.g., as an animal feed ingredient, for composting or even discarded on the landfills as waste. This results in the loss of valuable nutrients and, in the case of landfill, environmental pollution. Consequently, proper valorisation of pomace via its conversion into higher-value products is an important task, which may significantly increase the sustainability of the horticultural and fruit processing sectors. Ideally, the pomace should be upcycled by obtaining the highest added value ingredients and reaching the goal of zero waste [1].

Many small fruit species, which are commonly called berries, are particularly rich in polyphenolic antioxidants, natural pigments and various other bioactive compounds. Large fractions of berry constituents remain in the pomace after pressing the juice; in addition, the pomace contains dietary fibre, polyunsaturated fatty acid-rich oil, proteins and various micronutrients which are beneficial to health [2]. These constituents could be used in nutraceutical, cosmeceutical and functional food applications [3]. Therefore, the development of efficient recovery processes of such constituents from the pomace for nutritional and other human uses is an important and challenging task. Considering the heterogeneity of pomace, which consists of pulp, skins, seeds and some stems, different concepts may be used for its processing. Drying and milling is the simplest technology to produce pomace ingredients, while the recovery of the selected target groups of compounds [4] as well as their fractionation and purification requires more sophisticated techniques. For instance, during the last few years, various researchers have demonstrated techniques such as the consecutive extraction of pomace constituents with different polarities using supercritical fluid extraction with carbon dioxide (SFE-CO_2_), pressurized higher polarity liquids and, in some cases, enzymes, to raspberry [5], chokeberry [6], rowanberry [7] and other types of pomace. This approach enables researchers to recover lipophilic and higher polarity fractions, while the residues contain an increased content of insoluble dietary fibre and proteins. The products obtained may be used as high value ingredients for food, nutraceutical and cosmeceutical applications. Lipophilic berry pomace fractions mainly consist of PUFA-rich triacylglycerols (TAGs) and various bioactive compounds, like tocopherols, phytosterols and carotenoids, whose health benefits are well documented. For instance, EFSA has granted health claims to oleic, linoleic and α-linolenic acids and phytosterols for contributing to the maintenance of normal blood cholesterol levels, and to α-tocopherol (vitamin E) for contributing to the protection of cells from oxidative stress [8]. Therefore, there is great interest in the recovery and application of these lipid-soluble components for food, cosmetics and nutraceuticals. Moreover, considering the human applications of the recovered natural components, there is a clear tendency of using green, food- and environment-friendly technologies as alternatives to conventional extraction methods, particularly those that apply hazardous solvents. From this point of view, SFE-CO_2_ has been tested and commercialised for the extraction of lipophilic substances from various raw materials, including berries and their processing by-products [9,10,11]. The main advantages of using CO_2_ are its non-toxicity, low price and availability, as well as easy recycling and obtaining solvent-free extracts without energy consuming evaporation procedures. The dissolving ability of CO_2_ strongly depends on its pressure and temperature, which may be easily controlled. However, the high capital costs of SFE equipment are the main disadvantage of this technology; therefore, from a techno-economic assessment point of view, the method is more suitable for the extraction of high added value substances such as bioactive compounds beneficial to health.

In some cases, polar co-solvents such as ethanol may be added to the SFE-CO_2_ flow to adjust the solvent polarity and thereby increase the extraction yield and recovery rates of medium-polarity constituents [12]. In addition, mild extraction temperatures (40–70 °C) and an oxygen-free medium in SFE-CO_2_ processes supports the stability of oxidizable and thermally unstable constituents.

Different anatomical parts of rowanberries have been used in traditional medicines as natural medications for treating inflammatory, bacterial and viral diseases, tumours, diabetes and neurological and cardiovascular disorders [13]. Most of the studies on the chemical composition and properties of rowanberries have focused on their polyphenolic antioxidants [13,14], whereas only two articles have reported the isolation of lipophilic components from rowanberry pomace using SFE-CO_2_ [7,15]. For instance, Bobinaitė et al. (2020) optimized their extraction process for the highest yield conditions (45 MPa, 60 °C, 180 min) and obtained 4.8% of lipophilic rowanberry extract; however, the recovery of carotenoids was only up to 49.7% of the amount achieved by hexane extraction [7]. Other important lipophilic bioactive components were not reported.

In order to expand our knowledge on the use of SFE-CO_2_, both for the recovery of lipophilic rowanberry pomace constituents and their pre-fractionation, this study was undertaken with an aim to (1) use ethanol as a co-solvent to increase the yield of the extracts and individual groups of constituents, and (2) to evaluate the possibilities of increasing their concentration by using separators operating at subcritical parameters at different temperatures. To the best of our knowledge, only one study has previously applied this concept to berry pomace [16] by conducting the on-line SFE separation of cranberry pomace lipids using two separators. For the first separator, the temperature was kept below zero and pressure below the critical point (liquid state of CO_2_), while the second was depressurized at ambient conditions. The authors concluded that the concentration of various lipophilic compounds present in berry pomace could be increased due to the changes in their solubility in liquid CO_2_ at freezing temperatures. Considering remarkable compositional differences between the pomace, e.g., the highest yield of 4.8% for rowanberries [7] versus 11% for cranberries [17], it was of interest to test this concept for rowanberry pomace at previously optimized extraction conditions for the highest yield and fractionation at subcritical CO_2_ parameters. Thus, the hypothesis was that extraction with an ethanol co-solvent at supercritical parameters and fractionation at subcritical pressure and different temperatures below zero would enable us to pre-concentrate the selected groups of lipophilic compounds present in the rowanberry pomace. The results obtained may find practical application in the development of the specified ingredients for nutraceuticals.

## 2. Results and Discussion

### 2.1. The Yields of Extracts, Fractions and β-Carotene

The total yield of the extract is one of the most important process characteristics. Previously reported yields of lipophilic extracts recovered by SFE-CO_2_ from rowanberry pomace were comparatively low, up to 4.8%, while the recovery of carotenoids was less than 50% compared with hexane extraction [7]. Adding 5% ethanol (EtOH) as a co-solvent enabled previous researchers to increase the recovery of carotenoids from cranberry pomace (up to 66.25%) [16]. Therefore, in our study, several concentrations of co-solvent were applied (3, 5 and 7%) in order to increase the yield and the recovery of selected phytochemicals. In the preliminary experiments, the yield of the extract significantly increased by adding EtOH; from 5.55 (pure CO_2_) to 8.88 g/100 g (7% EtOH). Consequently, every 1% of added EtOH increased the total yield, on average, by 8.6%. The addition of a polar solvent effectively increased the solubility of higher polarity constituents and may have had an effect on the physical properties of the matrix, e.g., by enhancing the diffusion of the solutes from the solid particles.

The yields of the fractions were dependent on both the concentration of the co-solvent and the temperature in the first separator (further referred as S1). In general, the amount of the “lighter” fraction (LF), which was collected in the second separator (further referred as) S2, increased by decreasing the temperature in S1 from 0 to −20 °C and by increasing the amount of co-solvent. For operating parameters in S1 and S2, see Section 3.2. For instance, in the case of adding 7% EtOH, the yield of the LF was 1.9–2.1-fold higher than in case of 3% EtOH. The weight of the precipitates in the S1 extracts was not measured due to difficulties in its collection; however, it may be assumed that at higher EtOH concentrations and lower temperatures in S1, the ratio of “heavier” to “lighter” fractions (HF/LF) decreases. It is most likely that compounds extracted with the higher polarity solvent mixture, e.g., CO_2_ + 7% EtOH, remain more soluble in the solvents, even at the low temperatures in S1. A somewhat different result was obtained in the case of 5% EtOH, particularly during cooling S1 at −10 °C. It was observed that the yield amount of the LF was slightly higher than in the case of cooling S1 at −20 °C. The differences in the fraction yields provides a preliminary observation that fractionation may be performed by reducing the pressure and changing the temperature in S1. In addition, the redistribution of extract fractions also depends on the concentration of the co-solvent. It may be reasonably expected that the solubility of some of the extracted rowanberry pomace lipophilic constituents may reduce at freezing temperatures, particularly when adding EtOH. For instance, a well-known process of ‘winterization’ is used for the removal of waxes from crude EtOH extracts [18]. In general, the results obtained suggest that the compounds which are additionally solubilized by the added EtOH in the solvent mixture at supercritical CO_2_ conditions do not precipitate in S1 at freezing temperatures and are carried to S2.

The total extraction yield of lipophilic components is an important indicator of SFE-CO_2_ efficiency, but this is not the only important process criterion. When extracting ingredients for functional foods and nutraceuticals, the overall recovery of the target bioactive compounds from the raw material as well as their concentration in the extract may be even more important process characteristics.

Oil-soluble β-carotene is the most common form of carotene in plants. It is a red-orange pigment which can be used as a highly prized colorant by the food industry and is also a well-known bioactive compound (as a vitamin A precursor). Therefore, the recovery of β-carotene is an important indicator of extraction effectiveness.

Previously, the content of β-carotene was reported to be 629.1 mg/100 g [7] in the rowanberry pomace of mixed cultivars. In the current study, the β-carotene content was analysed in the SFE-CO_2_ lipophilic extracts separated by fractionation. Preliminary experiments (no fractionation) showed that adding EtOH increased the recovery of β-carotene, by approx. 4.2% for 1% of the added co-solvent. However, due to the increased total extract yield and dilution of β-carotene with other constituents, its concentration in the extracts reduced from 175.7 ± 0.8 mg/100 g (0% EtOH) to 168.7 ± 0.8, 166.1 ± 1.9 and 142.8 ± 2.5 mg/100 g with 3, 5 and 7% of EtOH, respectively.

In the case of fractionation, both decreasing the temperature in S1 and increasing the co-solvent concentration increased the concentration of β-carotene in S2 (LF) and decreased its concentration in S1 (HF). Thus, the concentration of β-carotene in the LF obtained with 7% EtOH at −20 °C (Table 1, 238.1 ± 0.6 mg/100 g) was 66.7% higher than the concentration that was recovered from the non-fractionated extract using the same co-solvent concentration (142.8 ± 2.5 mg/100 g). Moreover, a lower S1 temperature resulted in a higher recovery of β-carotene from the LF. Therefore, the highest β-carotene recovery of 60.8% (as compared with hexane extract) was found in the LF at −20 °C and with a maximum co-solvent concentration of 7%.

These results are consistent with a previous study which demonstrated that increasing the EtOH co-solvent concentration can remarkably increase the solubility of β-carotene in SFE-CO_2_ [19]. The previously reported distribution of β-carotene extracted from cranberry pomace by SFE-CO_2_ with 0 and 5% EtOH in the LF and HF using similar fractionation parameters was not so evident [16]. However, the concentration of β-carotene in the rowanberry pomace extracts obtained in our study was more than 200 times higher than in the cranberry extracts, and therefore the fractionation results may not be directly comparable. This is because remarkable differences in concentration may have a crucial effect on the solubility at various temperatures and co-solvent concentrations.

### 2.2. Composition of Fatty Acids and Triacylglycerols (TAGs)

It was determined that the main fatty acids in the pomace oil of sweet rowanberry pomace were linoleic acid (57.33 ± 1.36%), oleic acid (22.36 ± 0.57%) and palmitic acid (9.98 ± 0.52%). Other fatty acids exceeding 1% of the total content were stearic acid (1.92 ± 0.25%), lignoceric acid (1.46 ± 0.05%) and behenic acid (1.39 ± 0.12%). Bobinatė et al. (2020) reported a similar fatty acid composition in a rowanberry pomace CO_2_ extract, with linoleic (61.18%), oleic (22.25%) and palmitic (9.00%) acids being the major constituents [7]. Therefore, in terms of percentage content, TAGs containing linoleic (L), oleic (O) and palmitic (P) acids were the most abundant. The TAGs containing longer chain fatty acids, e.g., behenic (C21:0) and lignoceric (C24:0), were not identified due to the lack of reliable MS data.

Previous studies have reported that in the majority of TAG molecules, the fatty acids in the sn1 and sn3 positions are different, but one of these may be similar to the fatty acid in the sn2 position [20]. The composition of rowanberry TAGs agrees with this assumption (Table 2). Based on the previously reported results and their interpretation (Zeb & Murkovic, 2010 [21] and references therein), we can hypothesize that the majority of TAGs identified in rowanberry oil quantitatively contained linoleic acid in the sn2 position, while four of the TAGs might also contain linoleic acid in the sn1 or sn3 position. The major TAGs in rowanberry pomace oil were composed of unsaturated LLL and OLL; their contents were 34.93–36.13% and 26.52–27.87%, respectively (Table 2). The second largest group of TAGs (quantitatively), SLL and PLL, constituted 11.92–12.82% and 10.43–11.65%, respectively. TAGs containing all three different fatty acids, including saturated, mono and polyunsaturated fatty acids, were present at 3.09–6.49%, while the most highly saturated TAG (PLS) was the least abundant.

In general, the effect of a co-solvent on the TAG composition was negligible, although statistical data evaluation indicates an increase in the contents of LLLn, SLO, SLL and PLO and a decrease in LLL content when the co-solvent concentration was increased. The composition of TAGs in LF fractions obtained at different S1 temperatures was also quite similar. Some redistribution of TAGs may be expected due to the differences in melting points between more saturated and more unsaturated TAGs; however, it seems that the temperature applied in S1 did not have significant effect on the solubility of TAGs in subcritical (liquid) CO_2_. According to a previous study [16], the differences in the percentage composition of TAGs in the LF and HF of cranberry pomace were also not significant. To the best of our knowledge, this is the first report on the TAG composition of rowanberry oil.

### 2.3. Tocopherols and Phytosterols

Tocopherols are lipophilic antioxidants which can protect PUFAs from peroxidation [22]. In human nutrition, the daily administration of α-tocopherol (vitamin E) is essential, as it is the most effective lipophilic antioxidant in the human organism.

Three tocopherols (α, β and γ) and three phytosterols were identified and quantified in the total extracts and both fractions, LF and HF. The concentration of α-tocopherol was found to be up to 5.5-fold higher than that of β + γ-tocopherol under the same conditions. Preliminary evaluation (without fractionation) showed that adding 5 and 7% of EtOH significantly increased the total recovery of tocopherols from the rowanberry pomace. The highest recovery was obtained after adding 5% of EtOH, which agrees with the results of Tamkutė et al. (2020) [16] and Kraujalis & Venskutonis [23] on the SFE-CO_2_ extraction of cranberry pomace and amaranth, respectively. Thus, the average increase in the recovery of α and β + γ-tocopherols for each added 1% of EtOH was 3.5 and 0.84 µg/g of pomace, respectively. However, the effect of EtOH on the concentration of tocopherols in the extracts was not as evident due to the increase in the total yield and the consequent dilution of tocopherols with other extracted substances.

**Figure 1:**
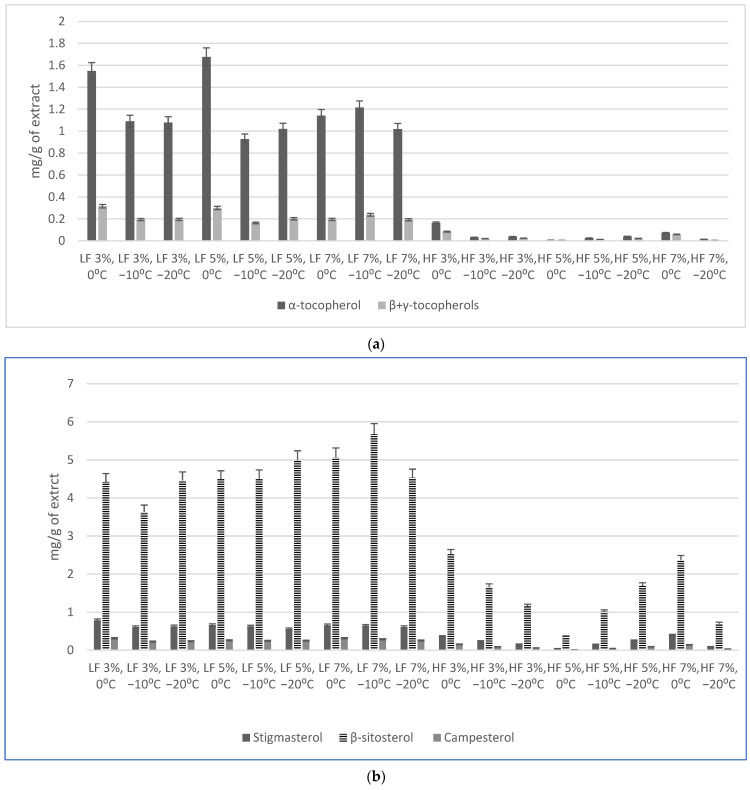
The concentration of (**a**) tocopherols (**b**) phytosterols (mg/g of extract) in the fractionated SFE-CO_2_ extracts; HF—heavier fraction; LF—lighter fraction. The bars represent mean values and standard deviations of 3 replicate measurements.

It is evident that the majority of the tocopherols was transferred to S2 (Figure 1a, LF); for instance, the concentration of α-tocopherol in the LF was at least 10-fold higher than in the HF. The highest concentration of α-tocopherol (1675 ± 136 µg/g) and β + γ-tocopherols (300.8 ± 2.8 µg/g) was found in the LF obtained using 5% EtOH at 0 °C in S1; the concentration was by 39% higher than in the total extract isolated under similar extraction parameters. Somewhat similar tendencies were observed in the extraction using 3% EtOH, while the differences in the concentrations of tocopherols in the LF at different temperatures in S1 were not so evident. Similar results were reported for cranberry pomace, in which the results were expressed in chromatogram peak area [16].

Phytosterols (plant origin sterols) constitute more than 250 various structures, while the most abundant are sitosterol, stigmasterol and campesterol. Phytosterols, as the precursors of plant growth factors, play an essential role in the function and structure of cell membranes, similarly to cholesterol in mammalian cells [24]. For patients with elevated blood cholesterol levels, the suggested daily dose of plant sterols would be 2 g as a food supplement [25]. In a previous study where rowanberry cuticular wax was extracted using chloroform and a mixture of hexane/ethyl acetate, the β-sitosterol content was 2.91 mg/g of extract [26].

In the current study, where the SFE-CO_2_ extraction of the rowanberry pomace was carried out with EtOH as a co-solvent with fractionation, the concentrations of β-sitosterol in the LF ranged from 3.63 mg/g to 5.67 mg/g, being remarkably higher than its concentration in the HF, 0.36 mg/g to 2.58 mg/g, respectively (Figure 1b). The concentrations of stigmasterol in the LF and HF were 0.56–0.78 and 0.09–0.39 mg/g, respectively, while the concentrations of campesterol were 0.23–0.31 and 0.03–0.16 mg/g, respectively. Consequently, the concentration of phytosterols was always higher in the LF than HF; however, the results obtained did not reveal any unambiguous tendencies regarding the effects of a co-solvent and temperature in S1.

### 2.4. Volatile Constituents

In total, 62 volatile compounds were identified in the headspace of different sweet rowanberry *cv Sahharnaja* pomace SFE-CO_2_ extracts, which accounted for 88.4–96.9% of the total integrated GC peak area. The constituents that were detected in at least three rowanberry extract samples are provided in Table 3, and the representative chromatograms and mass spectra of major (and a few other) compounds are presented in the Supplementary Materials. These show that the quantitative composition of volatiles is rather complex, consisting of aromatics, alcohols, acids, aldehydes, lactones, esters, terpenes, *n*-alkanes and other classes of compounds. Benzaldehyde and benzyl alcohol were the most abundant compounds released in the headspace of the SFE-CO_2_ extract. These results agree with previously reported data on the volatile composition of rowanberry fruits [27,28,29]. 2-Pentanone, benzaldehyde and methyl butyrate were found to be the major volatiles in aromatic extracts of rowanberry fruits [27], while benzaldehyde, (*E*)-2-hexenal and benzyl alcohol were selected by AEDA as principal aroma-active components [28]. In another study, benzaldehyde, benzyl alcohol and acetic acid were found in high amounts in alcohol extracts of dried rowanberries, whereas terpenoids and acids were determined at lower concentrations [29].

Other components whose contents exceeded 1% included 2-oxo-2-phenylacetonitrile (benzoyl cyanide, 0.06–3.27%), phenyl ethyl alcohol (1.09–2.52%), benzoic acid (0.19–2.57%), diethyl hydroxybutanoate (0.17–12.74%), furfural (0.19–2.06%), butyrolactone (0.30–1.57%), etc. (Table 3). In general, the volatile profile of the total extracts of the LFs was quite similar, although some variations were observed for the products obtained at different separation temperatures. For example, the percentage content of the major compound benzaldehyde in the total SFE-CO_2_ extracts ranged from 69.66 ± 2.32% (0% EtOH) to 82.89 ± 1.73% (3% EtOH). In the case of the LFs, the contents varied from 69.53 ± 0.96 (LF, 3% EtOH, −20 °C) to 87.75 ± 0.92% (LF, 5% EtOH, −20° C) (Table 3).

Although the highest content of benzaldehyde was determined at −20 °C with 5% EtOH, at lower EtOH concentrations the highest levels of benzaldehyde were obtained at the highest temperature (0 °C) used in the current study. The highest quantities of the other major volatile compounds, such as butyrolactone, furfural, benzyl alcohol and phenyl ethyl alcohol, were identified at −20 °C with 3% EtOH. However, for benzoic acid and 2-oxo-2-phenylacetonitrile, the most suitable temperatures with the same amount of EtOH were −10 °C and 0 °C, respectively.

Volatile compounds in the HFs were not analysed due to the insufficient amount of these fractions. However, based on the previously reported data for cranberry extracts, which demonstrated that their HFs were almost empty of volatiles, it may be reasonably assumed that the majority of the soluble rowanberry volatile compounds under the CO_2_ parameters in S1 will be transferred to the LF collection vessel (indicated as S2). It may be noted that some evaporation of the most volatile compounds may occur due to a boiling phenomenon, which can happen when pressure is reduced from 7 MPa and sub-zero temperatures (liquid CO_2_) to 0.1 MPa and ambient temperature (gaseous CO_2_). For instance, it is most likely that the EtOH co-solvent at the applied concentration almost fully evaporated from the final extract (i.e., the “lighter” fraction).

**Figure 2:**
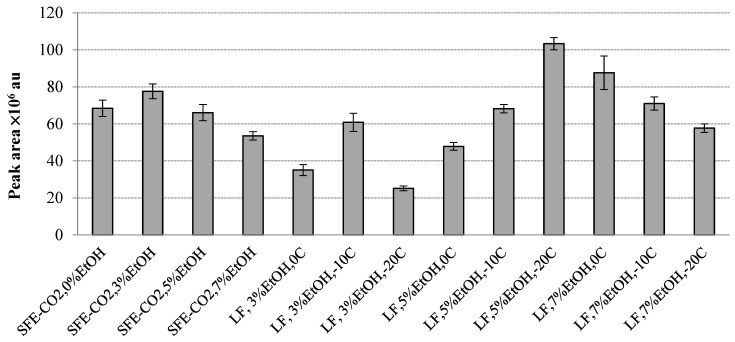
Benzaldehyde peak area integrated by GC×GC-TOF/MS in the headspace of the total rowanberry extracts (T) and its fraction (LF) at different EtOH concentrations (%) and separation temperatures in S1 (°C).

**Figure 3:**
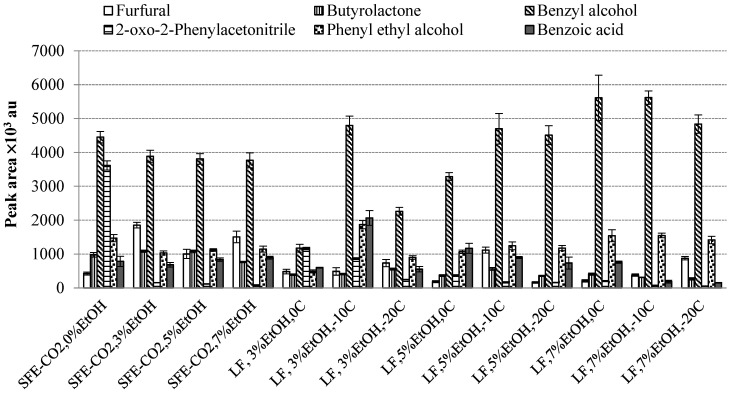
The peak areas of the major volatiles in the headspace of the total rowanberry extracts (T) and its fraction (LF) at different EtOH concentrations (%) and separation temperatures in S1 (°C).

Figure 2 illustrates the concentration of the most abundant volatile aromatic compound released into the headspace of the extracts, benzaldehyde, expressed in peak area units (au). Peak areas provide more comprehensive information about the extraction efficiency as long as they are directly related to the absolute concentration of the released volatiles. They can also be closely related to the sensory properties of rowanberry extracts obtained at different extraction conditions. Thus, the amounts of benzaldehyde in different rowanberry SFE-CO_2_ extracts were significantly different and varied from 25.18 ± 1.28 × 10^6^ au (LF, 3% EtOH, −20 °C) to 103.31 ± 1.28 × 10^6^ au (LF, 5% EtOH, −20 °C) (Figure 2). It may be assumed that as a major aromatic compound, benzaldehyde would play an important role in the overall rowanberry extract aroma; it possesses ”almond, burnt sugar” [31], “sharp, sweet, bitter almond, cherry” and “almond, fruity, powdery, nutty, cherry, maraschino cherry” odour notes [32]. Naturally, benzaldehyde has various edible plant origins, such as almonds, apricots, cherry kernels, apples, black tea, Chardonnay wine, etc. [33]. Benzaldehyde is also commonly employed to confer almond flavour to foods and scented products; moreover, it is used in cosmetics. As its intended use is in food, cosmetics, pharmaceuticals or soap, benzaldehyde is “generally regarded as safe” (GRAS) by the US FDA and FEMA. The absolute amounts of other quantitatively important volatile compounds are summarized in Figure 3.

Benzyl alcohol, as the second major volatile compound, is an aromatic alcohol which has been characterized as possessing “sweet, flower” [31],“berry, cherry, grapefruit, citrus, walnut” and “floral, rose, phenolic, balsamic, sweet, fruity and chemical” odour notes [32]. The percentage content of benzyl alcohol in different rowanberry SFE-CO_2_ extracts ranged from 2.61 ± 0.03% (LF, 3% EtOH, 0 °C) to 6.35 ± 0.03% (LF, 3% EtOH, −20 °C) (Table 3), while the concentration varied from 1.17 ± 0.11 × 10^6^ au (LF, 3%EtOH, 0 °C) to 5.61 ± 0.67 × 10^6^ au (LF, 7%EtOH, 0 °C) (Figure 3).

The percentage distribution of benzoic acid ranged from 0.19 to 2.57% (Table 3); the absolute amount varied from 154.4 ± 8.6 × 10^3^ au (LF, 7% EtOH, −20 °C) to 2.06 ± 0.22 × 10^6^ au (LF, 3% EtOH, −10 °C) (Figure 2). Naturally occurring benzoic acid is a strong antimicrobial agent; it has a balsamic, sweet, honey-like aroma and was reported to be among the aromatic contributors to cranberry vine, juice and pomace SFE-CO_2_ extracts [22].

Benzyl alcohol, benzaldehyde and benzoic acid, which were reaffirmed as generally recognized as safe (GRAS), are used in cosmetic formulations as preservatives and fragrance ingredients. The safety of benzyl derivatives is supported by the fact that their intake as natural food components is larger than that of intentional additions as flavouring substances [34]. It may be concluded that *S. aucuparia* pomace SFE-CO_2_ extracts and their fractions might be potential natural flavourings providing aromatic notes of benzaldehyde, benzyl alcohol and some other natural phytochemicals. 

## 3. Materials and Methods

### 3.1. Chemicals, Solvents and Gasses

CO_2_ and N_2_ were obtained from Gaschema (Jonava, Lithuania), EtOH from agricultural origin (96.6%) from MV Group Production (Kaunas, Lithuania), (±)—α-tocopherol, rac-β-tocopherol, (+)—γ-tocopherol, δ—tocopherol, β—carotene and a mixture of 37 fatty acid methyl esters (FAMEs) were obtained from Sigma-Aldrich (Steinheim, Germany).

### 3.2. Extraction and Fractionation of Rowanberry Pomace by SFE−CO_2_

The fruits of sweet rowanberry *cv* Sahharnaja were frozen and stored at −20 °C. After defrosting, they were pressed in a Smeg SJF01CREU Juicer (Smeg S.p.A, Guastalla, Italy); the pomace was lyophilized in an Advantage Plus Freeze Dryer (SP Industries, Warminster, PA, USA) for 72 h at 30 µbar, ground in a Retsch ZM200 mill (Haan, Germany) using a 0.5 mm sieve and stored at 18 °C. In our previous study, the moisture content of rowanberry pomace freeze-dried under similar conditions was 4.9% [7].

The SFE was conducted using 20 g of pomace in a Helix extractor (Applied Separation, Allentown, PA, USA) equipped with a 500 cm^3^ cell and two separators, S1 and S2. In this system, a pressure of 42.4 MPa in the extraction cell was adjusted in the main pump while a temperature of 53 °C was maintained by a surrounding heating jacket [7]. The pressure in S1 was controlled manually by the micro-metering valve installed on the line between the extraction cell and separator S1. In fact, S2 was used for collecting the non-precipitated S1 substances at atmospheric pressure. In this study, gas flow rate is reported in SL/min, which refers to litres per minute at standard temperature (20 °C) and pressure (101.3 kPa). This was measured by a flowmeter located after S1, and the flow rate was slightly fluctuating (approx. between 1.9–2.1 SL/min, on average 2 SL/min) due to some fluctuations in the manually controlled pressure after reducing it to 7 MPa. These parameters were applied for all SFE-CO_2_ experiments. The first fraction (which will be further referred to as heavier, HF) was precipitated by reducing the temperature in S1 to 0, −10 and −20 °C, while the pressure was maintained at 7 MPa. The densities of fluid CO_2_ at these parameters are 955.3, 1007 and 1053 kg/m^3^, respectively. The cooling of S1 was achieved by using a thermostatic bath and FT402 FT Immersion Cooler (JULABO GmbH, Seelbach, Germany). The second fraction (further referred to as lighter, LF), which remained soluble under liquid CO_2_ parameters, was collected from the depressurized S2 at room temperature. A co-solvent ethanol (EtOH) was introduced at concentrations of 0, 3, 5 and 7% and flow rate of 0.228 mL/min. The duration of every extraction was 2 h. Preliminary extraction results were tested at similar parameters without using separators. The extracts collected at the end of the process, after depressurizing the system at room temperature without using S1 for precipitating any insoluble fraction, are further reported as ‘non-fractionated extracts’. The fractions were kept at −20 °C.

### 3.3. Analysis of β-Carotene

Determination of the β-carotene concentration in the extracts was conducted chromatographically according to Zymone et al. [35] using a Waters 2695 liquid separation module (Water Corporation, Milford, MA, USA) and a UV–Vis detector (UV–Vis, 2489, Water Corporation, USA) to monitor the elution of compounds. β-carotene was quantified at 450 nm. Separation was conducted on a RP-C30 column (5 µm, 250 × 4.0 mm, YMC Europe, Dinslaken, Germany) integrated with a C30 guard column (5 µm, 10 × 4.0 mm, YMC Europe, Dinslaken, Germany). The eluent flow rate was 0.65 mL/min, and the temperature of the column was maintained at 25 °C. The mobile phase consisted of methanol (solvent A) and methyl tert-butyl ether (solvent B). All of the samples were injected at 40% B and held for 5 min. The gradient was changed to 83% B in 50 min, and then to 100% B in 5 min and held for 10 min. Finally, the gradient was changed to 40% B in 5 min and held for 10 min.

### 3.4. Determination of Fatty Acids and Triacylglycerols (TAGs)

Fatty acid composition was analysed using the Sukhija & Palmquist method [36]. Briefly, the lipids were extracted and methylated in a one-step procedure using toluene. Fatty acid methyl esters (FAMEs) were analysed on an Agilent 6890A GC (Agilent Technologies, Santa Clara, CA, USA) equipped with a flame ionization detector (FID) and capillary column with a liquid phase CP-Sil 88 (100 m × 0.25 mm, 0.20 μm). The results are expressed as the percentage content of individual acids over the sum of the total integrated GC peak area.

TAGs were analysed using the method of Zeb and Murkovic [21] using the Waters AQCUITY Ultra Performance Liquid Chromatography System (UPLC, Waters Corp., Milford, MA, USA) equipped with a quadrupole time-of-flight (QTOF) mass spectrometer (maXis 4G QTOF, Bruker Daltonics, Bremen, Germany) using an Acquity BEH, C18 column, 2.1 × 50 mm, particle size 1.7 µm (Waters, Wexford, Ireland). The autosampler and column oven temperatures were 20 °C and 40 °C, respectively. An isocratic solvent system consisting of 18% isopropanol in methanol (0.1% acetic acid) and 0.05% of ammonium acetate was used, with a 0.4 mL/min flow rate and 10 min separation time. Fragmentor’s potential was 150 V, capillary voltage 4000 V, drying gas temperature of 350 °C and *m*/*z* range 200–1000. Positive electrospray ionization (ESI) mode was used in the analysis and the TAGs were identified based on the presence of a protonated ion [M + H]^+^. The content of individual TAGs was expressed as the peak area percentage of the sum of TAG peak areas.

### 3.5. Determination of Tocopherols and Sterols

The method of Slavin and Yu [37] was used for saponifying sterol and tocopherol esters, which were analysed in the same system as the TAGs. The separation procedure started with 30% A (0.1% formic acid in water) and 70% B (0.1% formic acid in acetonitrile). In 1 min, B was increased to 100%, kept for 5 min and restored to the initial conditions in 0.5 min. The temperature was 30 °C and the flow rate 0.4 mL/min. Mass spectra were acquired using a QTOF mass spectrometer in the APCI positive ion mode, with an acquisition rate of 3 spectra per second in the mass range of 300 to 1200. The instrument was configured for time-of-flight (TOF) analysis. Other parameters were as follows: 2000 V of capillary voltage; 3000 nA of corona current; −500 V of endplate offset; 400 °C of vaporizer temperature; 1.6 bar of nebulizer gas pressure; 8 L/min of drying gas flow rate; and 200 °C of drying gas temperature. Protonated ion peaks [M + H]^+^ were used for quantification.

### 3.6. Evaluation of Volatile Aromatic Compounds 

The volatile compounds were analysed, identified and quantified using gas chromatography-time-off-flight mass spectrometry on a GC × GC-TOFMS LECO Pegasus 4D system, as described by Tamkutė et al. [16], with slight modifications. In this study, the primary oven was programmed as follows: 40 °C (1 min), ramped to 220 °C at 5 °C/min (1 min), finally ramped to 300 °C at 20 °C/min (hold 1 min). Other parameters were similar.

### 3.7. Statistical Data Evaluation

All analyses were carried out in triplicates using MS Excel 2016 for calculating mean values and standard deviations. One-way ANOVA was applied for determining significant differences using a Stat-graphics 18-X64 package. Tukey’s HSD was used to determine the significant difference among the treatments at *p* < 0.05.

## 4. Conclusions

The simultaneous separation of rowanberry pomace lipophilic compounds, isolated using SFE-CO_2_ with 3, 5 and 7% of a co-solvent (EtOH), enabled us to obtain two lipophilic products. These were conditionally referred to as the ‘heavier’ fraction (HF), which was collected in the first separator (S1) at 7 MPa and 0, −10 and −20 °C, and the ‘lighter’ fraction (LF), which was collected in the depressurized second separator (S2) and ambient temperature. This resulted in fractions with different concentrations of selected constituents. Every 1% of added co-solvent EtOH increased the total yield of the extract, on average, by 8.6%, while the yield of the LF increased by decreasing the temperature in S1 from 0 to −20 °C and by increasing the amount of co-solvent. EtOH increased the recovery of β-carotene by approx. 4.2% for every 1% of the added co-solvent; however, due to the increased total extract yield and dilution of β-carotene with other constituents, its concentration in the extracts reduced from 175.7 ± 0.8 (0% EtOH) to 142.8 ± 2.5 mg/100 g (7% EtOH). The decrease in the S1 temperature as well as the increase in co-solvent concentration increased the concentration of β-carotene in the LF and decreased its concentration in the HF; in the LF obtained at −20 °C, it was 66.7% higher than in the non-fractionated extract. The concentrations of phytosterols and tocopherols in the LF were also remarkably higher than in the HF; however, any clear dependence of the effect of a co-solvent concentration and S1 temperature on the compound concentration was not observed. Among 62 identified volatile compounds in the total rowanberry pomace SFE-CO_2_ extracts and their LF, the most abundant were benzaldehyde and benzyl alcohol, constituting up to 87.75 ± 0.92% (LF, 5% EtOH, −20°C) and up to 6.35 ± 0.03% (LF, 3% EtOH, −20 °C), respectively. The effect of a co-solvent and fractionation on quantitatively major lipophilic constituents, triacylglycerols and fatty acids, was negligible; the major TAGs of rowanberry pomace were composed of the major fatty acids oleic and linoleic LLL and OLL, forming 34.93–36.13% and 26.52–27.87% of TAGs, respectively.

In general, the results obtained in this study have proven our hypothesis regarding the possibility to pre-concentrate the selected groups of lipophilic compounds present in rowanberry pomace, by using the precipitation of insoluble compounds in the subcritical CO_2_ fraction in the separator installed after the main extractor, and cooling below 0 °C. Moreover, the use of an ethanol co-solvent may increase the total yield of the extracts and recovery of β-carotene. These findings enable researchers to upcycle fruit pomace in order to obtain beneficial ingredients, which may find applications in functional foods and nutraceuticals.

## Figures and Tables

**Table 1:** The concentration of β-carotene in rowanberry pomace extract fractions and its recovery from the LF.

EtOH, %	Separator S1 Temp, °C	Yield in LF, %	β-Carotene in LF, mg/100 g	β-Carotene Recovery in S2 with LF, mg/100 g Pomace	β-Carotene in HF, mg/100 g
3	0	1.87 ± 0.02 ^ab^	133.1 ± 2.2 ^a^	2.5	187.4 ± 0.1 ^l^
	−10	3.15 ± 0.10 ^cd^	174.1 ± 0.6 ^c^	5.5	48.94 ± 0.15 ^d^
	−20	3.61 ± 0.38 ^de^	213.3 ± 1.6 ^e^	7.7	52.38 ± 0.09 ^e^
5	0	2.92 ± 0.10 ^bcd^	145.1 ± 1.4 ^b^	4.2	131.5 ± 0.2 ^f^
	−10	5.53 ± 0.17 ^fgh^	219.0 ± 0.8 ^e^	12.1	17.82 ± 0.10 ^a^
	−20	4.51 ± 0.27 ^ef^	236.3 ± 1.2 ^f^	10.7	31.39 ± 0.18 ^b^
7	0	3.84 ± 0.37 ^de^	186.2 ± 1.5 ^d^	7.2	180.3 ± 0.1 ^k^
	−10	6.00 ± 0.42 ^gh^	178.2 ± 1.0 ^c^	10.7	nd
	−20	7.75 ± 0.25 ^ij^	238.1 ± 0.6 ^f^	18.5	37.41 ± 0.28 ^c^

HF—heavier fraction; LF—lighter fraction; nd—not determined; ^a–l^—different superscript letters within the same column indicate statistical differences (one-way ANOVA, *p* < 0.05).

**Table 2:** Composition of triacylglycerols (TAGs, %) of rowanberry pomace oil separated in S2 (LF).

No	TAG	0% EtOH	3% EtOH	5% EtOH	7% EtOH	Light Fraction (LF)								
						0 °C			−10 °C			−20 °C		
						3% EtOH	5% EtOH	7% EtOH	3% EtOH	5% EtOH	7% EtOH	3% EtOH	5% EtOH	7% EtOH
1	LLLn	1.76 ± 0.01 ^a^	1.59 ± 0.05 ^a^	1.64 ± 0.03 ^a^	1.65 ± 0.03 ^a^	1.74 ± 0.02 ^a^	1.62 ± 0.08 ^a^	1.74 ± 0.06 ^a^	1.71 ± 0.08 ^a^	1.62 ± 0.11 ^a^	1.69 ± 0.03 ^a^	1.63 ± 0.01 ^a^	1.62 ± 0.08 ^a^	1.57 ± 0.01 ^a^
2	SLO	3.09 ± 0.06 ^a^	3.18 ± 0.01 ^ab^	3.35 ± 0.14 ^ab^	3.66 ± 0.21 ^b^	3.24 ± 0.12 ^ab^	3.30 ± 0.05 ^ab^	3.49 ± 0.26 ^ab^	3.45 ± 0.01 ^ab^	3.38 ± 0.13 ^ab^	3.37 ± 0.11 ^ab^	3.36 ± 0.15 ^ab^	3.45 ± 0.06 ^ab^	3.43 ± 0.06 ^ab^
3	PLLn	1.04 ± 0.02 ^d^	0.92 ± 0.00 ^cd^	0.88 ± 0.05 ^bcd^	0.92 ± 0.09 ^cd^	0.92 ± 0.06 ^cd^	0.86 ± 0.04 ^abc^	0.78 ± 0.02 ^abc^	0.83 ± 0.04 ^abc^	0.83 ± 0.01 ^abc^	0.74 ± 0.02 ^ab^	0.79 ± 0.04 ^abc^	0.83 ± 0.04 ^abc^	0.70 ± 0.03 ^a^
4	PLS	1.74 ± 0.02 ^ab^	1.80 ± 0.02 ^ab^	1.67 ± 0.16 ^a^	2.15 ± 0.29 ^b^	1.70 ± 0.19 ^ab^	1.59 ± 0.05 ^a^	1.67 ± 0.06 ^a^	1.83 ± 0.06 ^ab^	1.55 ± 0.04 ^a^	1.75 ± 0.05 ^ab^	1.71 ± 0.14 ^ab^	1.70 ± 0.05 ^ab^	1.82 ± 0.01 ^ab^
5	OLL	26.77 ± 0.50 ^ab^	27.00 ± 0.08 ^abc^	27.44 ± 0.11 ^abc^	27.09 ± 0.30 ^abc^	26.52 ± 0.50 ^a^	27.05 ± 0.00 ^abc^	27.35 ± 0.43 ^abc^	27.17 ± 0.02 ^abc^	27.87 ± 0.00 ^c^	27.18 ± 0.15 ^abc^	27.76 ± 0.09 ^bc^	27.59 ± 0.27 ^bc^	27.08 ± 0.12 ^abc^
6	PLL	11.65 ± 0.09 ^d^	10.70 ± 0.04 ^ab^	10.71 ± 0.02 ^ab^	10.64 ± 0.25 ^ab^	11.42 ± 0.08 ^cd^	11.24 ± 0.40 ^bcd^	10.67 ± 0.02 ^ab^	11.02 ± 0.10 ^abcd^	10.79 ± 0.09 ^abc^	10.60 ± 0.06 ^ab^	10.67 ± 0.33 ^ab^	10.63 ± 0.02 ^ab^	10.43 ± 0.16 ^a^
7	LLL	35.26 ± 0.27 ^a^	36.13 ± 0.25 ^a^	35.52 ± 0.60 ^a^	34.93 ± 0.16 ^a^	35.57 ± 0.22 ^a^	35.57 ± 0.64 ^a^	35.68 ± 0.13 ^a^	35.01 ± 0.43 ^a^	34.88 ± 0.16 ^a^	36.01 ± 0.00 ^a^	35.62 ± 0.38 ^a^	35.28 ± 0.32 ^a^	36.12 ± 0.42 ^a^
8	SLL	11.92 ± 0.09 ^a^	12.54 ± 0.15 ^b^	12.55 ± 0.04 ^b^	12.62 ± 0.22 ^b^	12.54 ± 0.13 ^b^	12.35 ± 0.32 ^ab^	12.48 ± 0.06 ^b^	12.62 ± 0.15 ^b^	12.82 ± 0.00 ^b^	12.57 ± 0.07 ^b^	12.38 ± 0.13 ^ab^	12.79 ± 0.06 ^b^	12.72 ± 0.04 ^b^
9	PLO	6.49 ± 0.08 ^b^	6.13 ± 0.11 ^ab^	6.22 ± 0.05 ^ab^	6.35 ± 0.02 ^ab^	6.35 ± 0.08 ^ab^	6.43 ± 0.03 ^ab^	6.13 ± 0.03 ^ab^	6.36 ± 0.14 ^ab^	6.25 ± 0.14 ^ab^	6.07 ± 0.05 ^a^	6.06 ± 0.15 ^a^	6.11 ± 0.07 ^a^	6.12 ± 0.12 ^a^

L—linoleic acid; Ln—linolenic acid; O—oleic acid; P—palmitic acid; S—stearic acid. ^a–d^ Different letters within the same row indicate statistical differences (one-way ANOVA, *p* < 0.05).

**Table 3:** Quantitative amounts of major volatile compounds detected in the total and light fractions of SFE-CO_2_ rowanberry pomace extracts, peak area percentage (%).

No ^#^	Compound ^A^	RI-E	RI-L	Total				Light Fractions								
				SFE-CO_2_ + EtOH, %				SFE-CO_2_ + 3% EtOH			SFE-CO_2_ + 5% EtOH			SFE-CO_2_ + 7% EtOH		
				0	3	5	7	0	−10	−20	0	−10	−20	0	−10	−20
1	Furfural	832	828	0.38 ± 0.04	1.98 ± 0.07	1.17 ± 0.11	1.94 ± 0.18	1.07 ± 0.05	0.60 ± 0.09	2.06 ± 0.20	0.30 ± 0.05	1.28 ± 0.06	0.14 ± 0.01	0.19 ± 0.02	0.39 ± 0.04	1.06 ± 0.09
4	Butyrolactone	938	941	0.88 ± 0.02	1.16 ± 0.06	1.27 ± 0.04	1.00 ± 0.05	0.88 ± 0.04	0.51 ± 0.02	1.57 ± 0.05	0.56 ± 0.04	0.65 ± 0.02	0.30 ± 0.00	0.37 ± 0.02	0.32 ± 0.02	0.32 ± 0.04
18	Benzyl alcohol	1040	1031	4.02 ± 0.10	4.15 ± 0.08	4.47 ± 0.16	4.90 ± 0.25	2.61 ± 0.03	5.99 ± 0.19	6.35 ± 0.03	5.01 ± 0.12	5.39 ± 0.28	3.83 ± 0.08	5.12 ± 0.22	5.72 ± 0.09	5.83 ± 0.23
25	2-oxo-2-Phenylacetonitrile	1104	1095	3.27 ± 0.08	0.17 ± 0.01	0.14 ± 0.01	0.11 ± 0.01	2.60 ± 0.16	1.09 ± 0.06	0.69 ± 0.05	0.58 ± 0.03	0.19 ± 0.01	0.13 ± 0.00	0.19 ± 0.01	0.08 ± 0.00	0.06 ± 0.00
27	Phenyl ethyl alcohol	1119	1107	1.33 ± 0.02	1.11 ± 0.03	1.31 ± 0.06	1.49 ± 0.10	1.09 ± 0.01	2.33 ± 0.06	2.52 ± 0.02	1.63 ± 0.04	1.43 ± 0.07	1.00 ± 0.02	1.41 ± 0.07	1.58 ± 0.04	1.71 ± 0.10
33	Benzoic acid	1202	1197	0.71 ± 0.13	0.73 ± 0.08	0.98 ± 0.08	1.17 ± 0.04	1.32 ± 0.17	2.57 ± 0.05	1.55 ± 0.22	1.78 ± 0.21	1.03 ± 0.03	0.62 ± 0.14	0.70 ± 0.05	0.19 ± 0.04	0.19 ± 0.01
	Total identified, %			92.06	94.67	91.62	88.38	94.59	94.36	90.54	90.70	94.90	96.43	94.78	96.92	96.47

^#^ All detected volatile compounds are listed in the Supplementary Materials (Table S1) in the order of their elution from a nonpolar BPX-5 MS capillary column. ^A^ Identified on the basis of GC–TOF/MS spectra based on comparison with Adams [30], Nist, PubChem and Chemspider databases and calculated RI. RI-E, Retention indices calculated against C_7_–C_30_ *n*-alkanes on nonpolar BPX-5 MS column. RI-L, Retention indices on nonpolar DB-5 column reported in literature [30] or NIST (https://webbook.nist.gov (accessed on 10 February 2025)), PubChem (https://pubchem.ncbi.nlm.nih.gov (accessed on 10 February 2025)) and Chemspider (https://www.chemspider.com (accessed on 10 February 2025)) databases.

## Data Availability

The original contributions presented in this study are included in the article/Supplementary Materials. Further inquiries can be directed to the corresponding author.

## Supplementary Materials

The following supporting information can be downloaded at: https://www.mdpi.com/article/10.3390/molecules30040964/s1, Figure S1: Fragments of representative chromatograms comparing volatile constituents in the headspace of the total rowanberry pomace extracts (T) and its light fractions (LF) at different EtOH concentrations (%) and separation temperatures in S1 (°C), Figure S2: GC-MS identification data of the main and selected volatiles (mainly benzene derivatives) in the headspace of selected rowanberry pomace LF fractions, Table S1: Volatile compounds detected in the total and light fractions of SFE-CO_2_ rowanberry pomace extracts, peak area percentage (%).
