# Supplementary material for: Supercritical Fluid Extraction of Lipids from Rowanberry Pomace with Pure CO2 and Its Mixtures with Ethanol Followed by the On-Line Separation of Fractions
Source: Molecules. 2025 Feb 19;30(4):964. doi: 10.3390/molecules30040964 (PMC11858718; doi:10.3390/molecules30040964)
Supplement: Supplementary file 1 [file molecules-30-00964-s001.zip › molecules-3440976-supplementary.docx]

**Supplementary material**

Supercritical Fluid Extraction of Lipids from Rowanberry Pomace with Pure CO_2_ and Its Mixtures with Ethanol Followed by the On-Line Separation
of Fractions

Viive Sarv ^1,2^, Rajeev Bhat ^2^, Laura Jūrienė ^3^, Renata Baranauskienė ^3^, Dalia Urbonavičienė ^4^, Pranas Viškelis ^4^ and Petras Rimantas Venskutonis ^3,^*

^1^ Polli Horticultural Research Centre, Institute of Agricultural and Environmental Sciences, Estonian
University of Life Sciences, 69108 Viljandi, Estonia; viive.sarv@emu.ee

^2^ ERA-Chair for Food (By-) Products Valorisation Technologies (VALORTECH), Estonian University of Life Sciences, 51006 Tartu, Estonia; rajeev.bhat@emu.ee

^3^ Department of Food Science and Technology, Kaunas University of Technology,
LT-50254 Kaunas, Lithuania; laura.juriene@ktu.lt (L.J.); renata.baranauskiene@ktu.lt (R.B.)

^4^ Biochemistry and Technology Laboratory, Institute of Horticulture LAMMC, LT-54333 Kaunas, Lithuania; dalia.urbonaviciene@lammc.lt (D.U.); pranas.viskelis@lammc.lt (P.V.)

***** Correspondence: rimas.venskutonis@ktu.lt


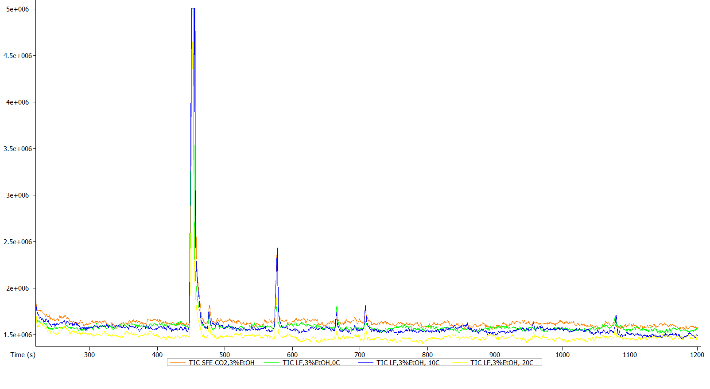

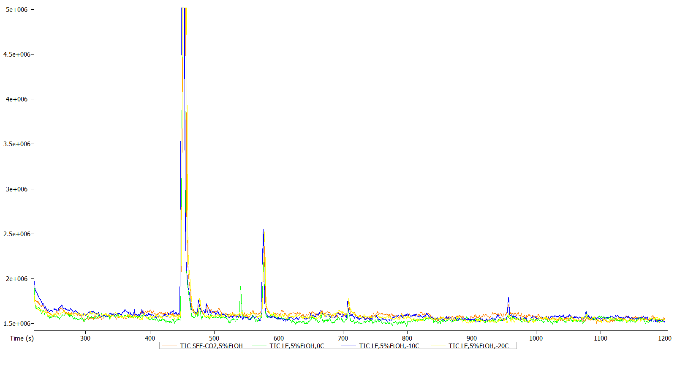


(A)

(B)


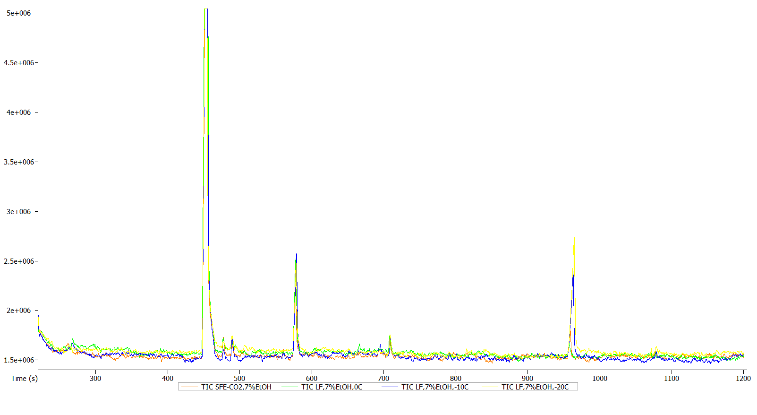


benzyl alcohol

benzaldehyde

(C)

**Figure S1.** Fragments of representative chromatograms comparing volatile constituents in the headspace of the total rowanberry pomace extracts (T) and its light fractions (LF) at different EtOH concentrations (%) and separation temperatures in S1 (° C): (A) SFE-CO_2_, 3% EtOH (orange), LF, 3% EtOH,0°C (green), LF, 3% EtOH,-10°C (blue), LF, 3% EtOH, -20 °C (yellow); (B) SFE-CO_2_, 5% EtOH (orange), LF, 5% EtOH,0°C (green), LF, 5% EtOH,-10°C (blue), LF, 5% EtOH, -20 °C (yellow); (C) SFE-CO_2_, 7% EtOH (orange), LF,7% EtOH,0°C (green), LF,7% EtOH,-10°C (blue), LF,7%EtOH, -20 °C (yellow).


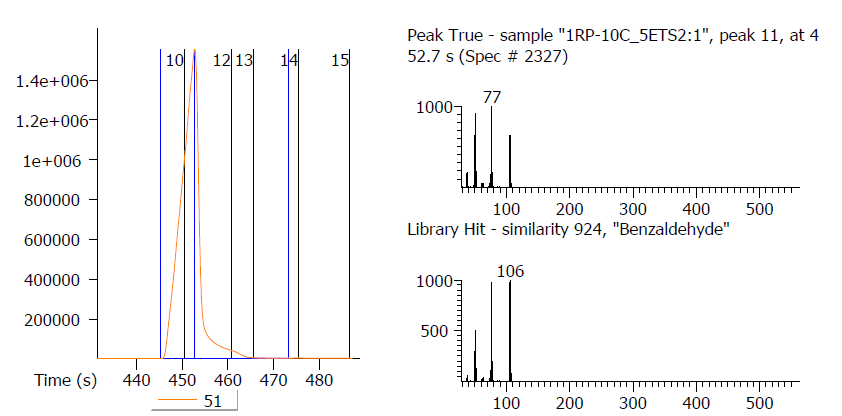

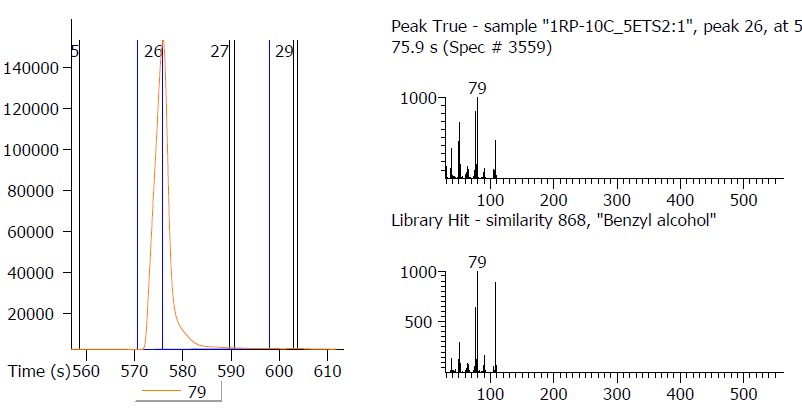


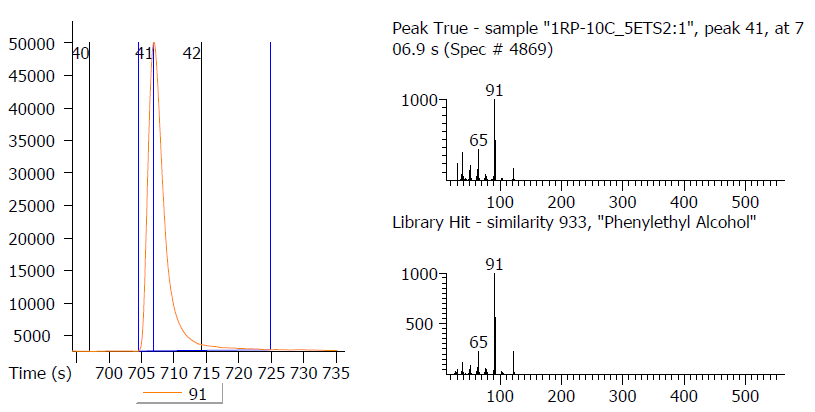

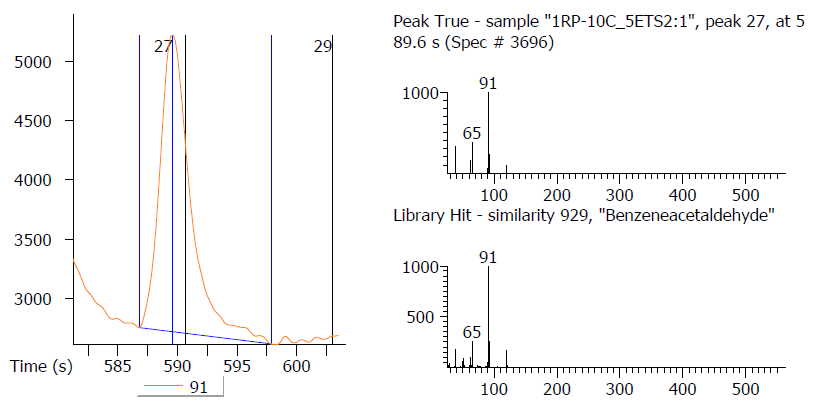


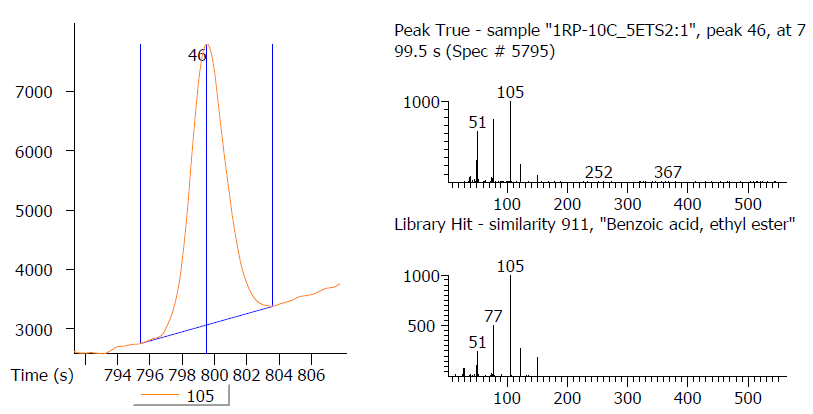

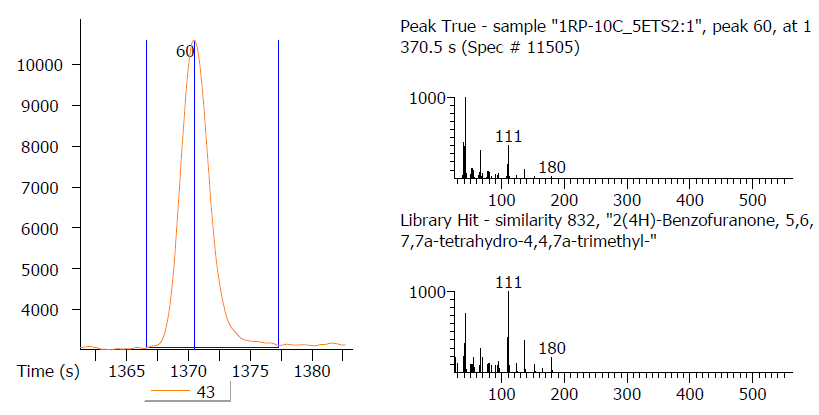


**Figure S2.** GC-MS identification data of the main and selected volatiles (mainly benzene derivatives) in the headspace of selected rowanberry pomace LF fractions

**Table S1.** Volatile compounds detected in the total and light fractions of SFE-CO_2_ rowanberry pomace extracts, peak area percentage (%)

|  | Compound ^A,^* | RI-E | RI-L | Total | | | | Light fractions | | | | | | | | |
| --- | --- | --- | --- | --- | --- | --- | --- | --- | --- | --- | --- | --- | --- | --- | --- | --- |
|  |  |  |  | SFE-CO_2_ + EtOH,% | | | | SFE-CO_2_ + 3% EtOH | | | SFE-CO_2_ + 5% EtOH | | | SFE-CO_2_ + 7% EtOH | | |
|  |  |  |  | 0 | 3 | 5 | 7 | 0 | -10 | -20 | 0 | -10 | -20 | 0 | -10 | -20 |
| 1 | Furfural | 832 | 828 | 0.38±0.04 | 1.98±0.07 | 1.17±0.11 | 1.94±0.18 | 1.07±0.05 | 0.60±0.09 | 2.06±0.20 | 0.30±0.05 | 1.28±0.06 | 0.14±0.01 | 0.19±0.02 | 0.39±0.04 | 1.06±0.09 |
| 2 | 2-Methyl butanoic acid | 846 | 841 | 0.16±0.01 | 0.09±0.01 | 0.12±0.01 | 0.15±0.01 |  | 0.12±0.01 |  |  | 0.11±0.00 | 0.09±0.00 | 0.10±0.01 | 0.09±0.00 |  |
| 3 | Heptanal | 905 | 901 | 0.33±0.01 |  |  |  | 0.36±0.01 |  |  | 0.12±0.02 |  |  |  |  |  |
| 4 | Butyrolactone | 938 | 941 | 0.88±0.02 | 1.16±0.06 | 1.27±0.04 | 1.00±0.05 | 0.88±0.04 | 0.51±0.02 | 1.57±0.05 | 0.56±0.04 | 0.65±0.02 | 0.30±0.00 | 0.37±0.02 | 0.32±0.02 | 0.32±0.04 |
| 5 | *(2E)*-Heptenal | 949 | 945 | 0.88±0.02 |  | 0.74±0.00 | 0.10±0.01 | 0.58±0.07 | 0.16±0.02 |  | 0.15±0.01 |  |  | 0.09±0.00 |  |  |
| 6 | γ-Valerolactone | 951 | 948 |  |  | 0.14±0.02 |  |  |  |  | 0.17±0.01 | 0.14±0.01 |  |  |  |  |
| 7 | Ethyl cyanacetate | 960 | 959 |  |  |  | 0.07±0.01 |  |  |  |  |  |  | 0.13±0.01 | tr |  |
| 8 | Benzaldehyde | 961 | 960 | 71.60±1.32 | 82.89±1.73 | 77.39±0.49 | 69.66±2.32 | 78.08±0.71 | 75.90±1.24 | 70.73±0.90 | 72.92±2.31 | 78.44±0.79 | 87.75±0.92 | 79.99±0.82 | 72.18±1.99 | 69.53±0.96 |
| 9 | δ-Valerolactone | 965 | 965 |  | 0.17±0.02 | 0.21±0.01 | 0.17±0.01 | 0.14±0.00 | 0.12±0.01 |  |  | 0.09±0.01 |  | 0.07±0.01 | 0.07±0.01 | 0.07±0.00 |
| 10 | 6-Methyl-5-hepten-2-one | 985 | 984 | 1.80±0.03 |  | 0.13±0.03 |  |  |  |  | 0.14±0.00 | 0.30±0.01 | 0.31±0.02 | 0.32±0.01 |  |  |
| 11 | *n*-Octanal | 999 | 998 | 0.04±0.01 |  |  |  |  |  |  |  |  |  |  | 0.08±0.01 | 0.10±0.00 |
| 12 | Hexanoic acid | 1000 | 998 | 0.71±0.04 | 0.13±0.01 | 0.15±0.00 | 0.17±0.01 | 1.83±0.02 | 0.36±0.01 | 0.48±0.02 | 0.70±0.03 | 0.50±0.06 | 0.16±0.01 |  | 0.22±0.01 | 0.18±0.01 |
| 13 | Diethyl oxalate | 1002 | 998 |  | 0.13±0.02 | 0.37±0.02 | 1.29±0.05 |  | 0.20±0.01 |  | 0.15±0.09 |  | 0.11±0.00 |  | 1.83±0.06 | 1.36±0.06 |
| 14 | Ethyl hexanoate | 1005 | 1003 |  | 0.05±0.01 | 0.06±0.00 | 0.08±0.01 | 0.07±0.00 | tr |  | 0.10±0.01 | 0.12±0.00 | tr | 0.08±0.00 | 0.09±0.00 | tr |
| 15 | *(2E,4E)*-Heptadienal | 1009 | 1007 | 0.07±0.00 | 0.05±0.00 |  | 0.09±0.01 | 0.12±0.01 | 0.21±0.03 |  |  | 0.07±0.00 | 0.06±0.00 | 0.08±0.01 | 0.08±0.00 | 0.07±0.00 |
| 16 | 2H-Pyran-2,6(3H)-dione | 1011 | - |  | 0.25±0.01 | 0.28±0.01 | 0.39±0.02 |  | 0.30±0.01 | 0.34±0.02 | 0.24±0.04 | 0.30±0.01 | 0.17±0.01 | 0.24±0.03 | 0.44±0.02 | 0.42±0.02 |
| 17 | 2,2,6-trimethylcyclo- hexanone | 1033 | 1027 |  | 0.35±0.02 | 0.38±0.09 | 0.42±0.03 | 0.20±0.01 | 0.18±0.02 | 0.46±0.03 | 0.25±0.01 | 0.15±0.00 | 0.12±0.04 | 0.19±0.02 | 0.26±0.05 | 0.16±0.03 |
| 18 | Benzyl alcohol | 1040 | 1031 | 4.02±0.10 | 4.15±0.08 | 4.47±0.16 | 4.90±0.25 | 2.61±0.03 | 5.99±0.19 | 6.35±0.03 | 5.01±0.12 | 5.39±0.28 | 3.83±0.08 | 5.12±0.22 | 5.72±0.09 | 5.83±0.23 |
| 19 | Pantolactone | 1042 | 1032 |  |  | 0.08±0.02 | 0.07±0.02 |  |  |  |  | 0.08±0.00 | tr | 0.05±0.01 | 0.07±0.00 | 0.08±0.00 |
| 20 | Benzene acetaldehyde | 1042 | 1036 |  | 0.05±0.00 | 0.06±0.00 | 0.09±0.01 |  | 0.13±0.01 |  |  | 0.08±0.00 | 0.05±0.00 | 0.05±0.00 | 0.09±0.01 | 0.08±0.00 |
| 21 | 3-Methyl-2-cyclohexen-1-one | 1049 | 1046 | 0.95±0.03 | 0.21±0.01 | 0.24±0.01 | 0.37±0.02 | 0.28±0.02 | 0.17±0.01 | 0.25±0.03 | 0.19±0.01 | 0.16±0.01 | 0.10±0.03 | 0.16±0.01 | 0.18±0.01 | 0.12±0.01 |
| 22 | (2E)-Octenal | 1052 | 1049 | 0.93±0.02 | 0.13±0.01 | 0.15±0.00 | 0.28±0.04 | 0.33±0.02 | 0.14±0.01 | 0.26±0.02 |  |  |  | 0.14±0.01 | 0.15±0.01 | 0.11±0.01 |
| 23 | Ethyl levulinate | 1078 | 1070 |  |  |  | 0.25±0.04 |  |  |  |  | 0.07±0.01 |  |  | 0.12±0.01 | 0.15±0.00 |
| 24 | Ethyl diethoxyacetate | 1096 | 1092 |  |  | 0.20±0.01 | 0.07±0.01 |  |  |  | 0.41±0.02 | 0.54±0.03 | 0.25±0.01 |  | 0.17±0.01 | 0.15±0.01 |
| 25 | 2-oxo-2-Phenylacetonitrile | 1104 | 1095 | 3.27±0.08 | 0.17±0.01 | 0.14±0.01 | 0.11±0.01 | 2.60±0.16 | 1.09±0.06 | 0.69±0.05 | 0.58±0.03 | 0.19±0.01 | 0.13±0.00 | 0.19±0.01 | 0.08±0.00 | 0.06±0.00 |
| 26 | *n*-Nonanal | 1108 | 1100 | 0.14±0.01 | 0.07±0.00 | 0.08±0.00 | 0.13±0.01 | 0.13±0.01 | 0.14±0.01 | 0.15±0.01 | 0.17±0.01 | 0.07±0.00 | 0.06±0.00 | 0.09±0.00 | 0.11±0.00 |  |
| 27 | Phenyl ethyl alcohol | 1119 | 1107 | 1.33±0.02 | 1.11±0.03 | 1.31±0.06 | 1.49±0.10 | 1.09±0.01 | 2.33±0.06 | 2.52±0.02 | 1.63±0.04 | 1.43±0.07 | 1.00±0.02 | 1.41±0.07 | 1.58±0.04 | 1.71±0.10 |
| 28 | 2-Ethyl hexanoic acid | 1132 | 1119 |  |  |  |  | 0.30±0.02 | 0.14±0.00 | 0.28±0.00 |  | 0.19±0.02 | 0.08±0.01 |  |  |  |
| 29 | Benzyl acetate | 1166 | 1157 | 0.07±0.00 | 0.12±0.00 | 0.14±0.01 | 0.17±0.01 |  | 0.11±0.01 | 0.19±0.01 | 0.12±0.00 | 0.14±0.00 | 0.12±0.00 | 0.13±0.01 | 0.18±0.01 | 0.19±0.00 |
| 30 | Ethyl benzoate | 1182 | 1169 | tr | 0.07±0.00 | 0.12±0.01 | 0.17±0.01 | 0.07±0.01 | tr | 0.08±0.00 | 0.08±0.01 | 0.13±0.00 | 0.05±0.00 | 0.09±0.01 | 0.20±0.00 | 0.27±0.01 |
| 31 | Diethyl succinate | 1190 | 1176 |  |  |  | 0.10±0.00 |  |  |  |  | tr |  |  | 0.21±0.00 | 0.32±0.02 |
| 32 | Octanoic acid | 1200 | 1190 |  |  |  |  | 0.10±0.02 |  |  | tr | 0.05±0.01 |  | tr |  |  |
| 33 | Benzoic acid | 1202 | 1197 | 0.71±0.13 | 0.73±0.08 | 0.98±0.08 | 1.17±0.04 | 1.32±0.17 | 2.57±0.05 | 1.55±0.22 | 1.78±0.21 | 1.03±0.03 | 0.62±0.14 | 0.70±0.05 | 0.19±0.04 | 0.19±0.01 |
| 34 | β-Cyclocitral | 1230 | 1217 | 0.30±0.00 | 0.06±0.01 | 0.07±0.01 | 0.10±0.02 |  | 0.11±0.00 |  | 0.09±0.02 | 0.05±0.01 | 0.05±0.01 | 0.06±0.01 | 0.08±0.01 | 0.09±0.00 |
| 35 | Ethyl phenylacetate | 1255 | 1243 |  |  | 0.05±0.00 | 0.08±0.01 |  |  |  |  | tr |  |  | 0.11±0.00 | 0.16±0.01 |
| 36 | Diethyl hydroxybutanoate | 1256 | 1244 |  | 0.17±0.01 | 0.60±0.02 | 2.45±0.19 |  | 0.52±0.03 | 0.70±0.05 | 0.45±0.04 | 1.48±0.04 | 0.38±0.04 |  | 6.94±0.08 | 12.74±0.58 |
| 37 | 2-Phenyl ethyl acetate | 1257 | 1254 | tr | 0.07±0.01 | 0.07±0.00 | 0.09±0.01 |  | 0.12±0.01 | 0.15±0.00 | 0.13±0.01 | 0.08±0.00 | 0.05±0.00 | 0.07±0.00 | 0.08±0.00 | 0.08±0.01 |
| 38 | Nonanoic acid | 1272 | 1267 |  |  |  |  | 0.21±0.01 |  |  | 0.13±0.00 |  |  | 0.09±0.01 |  |  |
| 39 | α-Cyanobenzyl alcohol | 1312 | 1301 | 0.14±0.02 | 0.07±0.00 | 0.10±0.01 | 0.21±0.02 | 0.82±0.12 | 0.89±0.03 | 0.64±0.05 | 0.63±0.10 | 0.36±0.05 | 0.09±0.02 | 0.15±0.02 | 0.20±0.02 | 0.30±0.03 |
| 40 | Geranyl acetone | 1453 | 1453 | 0.17±0.02 |  | 0.06±0.00 | 0.09±0.01 | 0.15±0.01 | 0.15±0.01 | 0.07±0.02 | 0.11±0.01 |  | 0.05±0.00 |  | 0.07±0.01 | 0.09±0.01 |
| 41 | *(E)*-β-Ionone | 1488 | 1487 | 0.30±0.04 | 0.06±0.00 | 0.08±0.00 | 0.11±0.01 | 0.18±0.01 | 0.18±0.01 | 0.25±0.01 | 0.14±0.01 | 0.07±0.00 | 0.07±0.01 | 0.05±0.00 | 0.08±0.00 | 0.11±0.01 |
| 42 | Dihydroactinidiolide | 1551 | 1539 | 0.51±0.03 | 0.18±0.02 | 0.21±0.01 | 0.27±0.03 | 0.73±0.06 | 0.58±0.03 | 0.76±0.05 | 0.49±0.04 | 0.22±0.01 | 0.18±0.02 | 0.15±0.01 | 0.20±0.01 | 0.25±0.02 |
|  | Total identified, % |  |  | 92.06 | 94.67 | 91.62 | 88.38 | 94.59 | 94.36 | 90.54 | 90.70 | 94.90 | 96.43 | 94.78 | 96.92 | 96.47 |

Compounds are listed in order of their elution from nonpolar BPX-5 MS capillary column.

* 3-Methyl butanoic acid, 2-heptanone, pentanoic acid, α-pinene, 4-methyl-pent-2-enolide, 1-octen-3-ol, myrcene, 2-pentyl furan, decane, *p*-cymene, limonene, γ-terpinene, 5,6-dihydro-2H-pyran-2-one, γ-hexalactone, *(Z)*-linalool oxide, *(E,E)*-3,5-octadien-2-one, γ-ethoxy butyrolactone, dodecane, ethyl octanoate and β-Ionone epoxide were detected only in one or two samples at the percentage concentration 0.04 - 0.80%.

^A^ Identified on the basis of GC–TOF/MS spectra based on comparison with Adams, Nist, PubChem and Chemspider databases and calculated RI.

RI-E, Retention indices calculated against C_7_–C_30_ *n*-alkanes on nonpolar BPX-5 MS column.

RI-L, Retention indices on nonpolar DB-5 column reported in literature^30^or Nist (https://webbook.nist.gov), PubChem (https://pubchem.ncbi.nlm.nih.gov) and Chemspider (https://www.chemspider.com) databases.
